# Aggregation of lipid rafts activates c-met and c-Src in non-small cell lung cancer cells

**DOI:** 10.1186/s12885-018-4501-8

**Published:** 2018-05-30

**Authors:** Juan Zeng, Heying Zhang, Yonggang Tan, Cheng Sun, Yusi Liang, Jinyang Yu, Huawei Zou

**Affiliations:** The First Oncology Department, Shengjing Hospital affiliated with China Medical University, Shenyang, 110004 China

**Keywords:** Lipid rafts, Mesenchymal-epithelial transition factor (c-met), C-Src, Radiation resistance, NSCLC

## Abstract

**Background:**

Activation of c-Met, a receptor tyrosine kinase, induces radiation therapy resistance in non-small cell lung cancer (NSCLC). The activated residual of c-Met is located in lipid rafts (Duhon et al. Mol Carcinog 49:739-49, 2010). Therefore, we hypothesized that disturbing the integrity of lipid rafts would restrain the activation of the c-Met protein and reverse radiation resistance in NSCLC. In this study, a series of experiments was performed to test this hypothesis.

**Methods:**

NSCLC A549 and H1993 cells were incubated with methyl-β-cyclodextrin (MβCD), a lipid raft inhibitor, at different concentrations for 1 h before the cells were X-ray irradiated. The following methods were used: clonogenic (colony-forming) survival assays, flow cytometry (for cell cycle and apoptosis analyses), immunofluorescence microscopy (to show the distribution of proteins in lipid rafts), Western blotting, and biochemical lipid raft isolation (purifying lipid rafts to show the distribution of proteins in lipid rafts).

**Results:**

Our results showed that X-ray irradiation induced the aggregation of lipid rafts in A549 cells, activated c-Met and c-Src, and induced c-Met and c-Src clustering to lipid rafts. More importantly, MβCD suppressed the proliferation of A549 and H1993 cells, and the combination of MβCD and radiation resulted in additive increases in A549 and H1993 cell apoptosis. Destroying the integrity of lipid rafts inhibited the aggregation of c-Met and c-Src to lipid rafts and reduced the expression of phosphorylated c-Met and phosphorylated c-Src in lipid rafts.

**Conclusions:**

X-ray irradiation induced the aggregation of lipid rafts and the clustering of c-Met and c-Src to lipid rafts through both lipid raft-dependent and lipid raft-independent mechanisms. The lipid raft-dependent activation of c-Met and its downstream pathways played an important role in the development of radiation resistance in NSCLC cells mediated by c-Met. Further studies are still required to explore the molecular mechanisms of the activation of c-Met and c-Src in lipid rafts induced by radiation.

**Electronic supplementary material:**

The online version of this article (10.1186/s12885-018-4501-8) contains supplementary material, which is available to authorized users.

## Background

Radiotherapy alone or combined with chemotherapy is the foundation for treating various solid tumors. However, radiation resistance greatly limits the curative effect of radiotherapy, which becomes one of the most important reasons for local recurrence and metastasis. Therefore, reversing the resistance of radiotherapy and increasing the radiosensitivity become the toughest challenge in cancer treatment.

Lipid rafts are special microdomains in the plasma membrane that influence cell proliferation, apoptosis, angiogenesis, immunity, cell polarity, and membrane fusion [1, 2]. c-Met, a receptor tyrosine kinase located in lipid rafts, promotes cancer cell migration and invasion and mediates resistance to current anticancer therapies, including radiotherapy. Studies have demonstrated that the activated residual of c-Met is located in lipid rafts [3, 4]. c-Src, a type of non-receptor tyrosine kinase, plays a vital role in a number of diverse cell signaling pathways, including cellular proliferation, cell cycle control, apoptosis, tumor progression, metastasis, and angiogenesis [5]. c-Src participates in radiation resistance [6] and might be the bridge to the activation of the downstream signaling pathway of c-Met. Whether and how lipid rafts are involved in the radio-resistance of non-small cell lung cancer (NSCLC) mediated by c-Met has not been established. We reveal here that disturbing lipid raft integrity inhibits the activation of c-Met and its downstream pathways, increases the sensitivity of NSCLC cells to radiotherapy, enhances the therapeutic ratio, and thus provides a new strategy to address the radio-resistance of NSCLC cells.

## Methods

### Cell lines, reagents and instruments

Human NSCLC cell line A549 (catalogue number: TCHu150) was obtained from the Cell Bank of the Chinese Academy of Sciences and H1993 (catalogue number: ATCC®CRL-5909™) was obtained from the American Type Culture Collection (ATCC). Methyl-β-cyclodextrin (MβCD) was purchased from Meilun Biotechnology (Dalian, Liaoning, China). Antibodies against c-Met, c-Src and β-actin were purchased from Wanlei Biotechnology (Shenyang, Liaoning, China). Antibodies against phosphorylated (p)-c-Met and p-c-Src were obtained from Bioss Inc. (Woburn, Massachusetts, USA). Anti-flotillin-1 antibody was obtained from Boster Biotechnology (Pleasanton, CA, USA). Fluorescein isothiocyanate-conjugated-anti-cholera toxin subunit B was purchased from Sigma (St. Louis, Missouri, USA). Horseradish peroxidase-conjugated specific goat anti-rabbit secondary antibody, Cy3-labeled goat anti-rat c-Met antibody, Cy3-labeled goat anti-rat c-Src antibody, phenylmethanesulfonyl fluoride (PMSF), radioimmunoprecipitation assay (RIPA) lysis buffer, SDS, trypsin and a cell cycle analysis kit were purchased from Beyotime Biotechnology (Shanghai, China). A cell apoptosis analysis kit was purchased from Nanjing Keygen Biotechnology (Nanjing, Jiangsu, China).

The following instruments were used: a linear particle accelerator used for human radiotherapy (Clinac 600C/D; ONCOR-PLUS, Siemens, Germany); a flow cytometer (C6; BD Biosciences, Franklin lakes, New Jersey, USA); a low-temperature refrigerated centrifuge (H-2050R; Xiangyi Company, Changsha, Hunan, China); a dual-gel vertical protein electrophoresis apparatus (DYCZ-24DN; Beijing Liuyi Biotech, Beijing, China); a gel imaging system (WD-9413B; Beijing Liuyi Biotech, Beijing, China); a fluorescence microscope (BX3; Olympus, Japan); and a Beckman SW40 rotor (Beckman Coulter GmbH, Unterschleissheim-Lohhof, Germany).

### Cell culture and treatment

A549 and H1993 cells were cultured in DMEM supplemented with 10% fetal bovine serum (FBS) at 37 °C under 5% carbon dioxide conditions. Cells were routinely subcultured in a monolayer, digested with 0.25% trypsin and stopped with DMEM when the cells covered 90% of the culture bottle. Then, the cells were cultured in FBS-free medium for another 24 h and prepared for various treatments.

MβCD is a cyclic polysaccharide containing a hydrophobic cavity that enables the extraction of cholesterol from cell membranes [7]. Cholesterol is the main component of lipid rafts. Therefore, MβCD is widely used as a lipid raft inhibitor. In this study, MβCD was dissolved in DMEM and used at final concentrations of 5 and 10 mM. In the experimental groups, cells were pretreated with MβCD for 1 h before irradiation. Control cells were treated with equal volumes of DMEM. As previous studies have shown, the survival fraction of A549 cells decreases when treated with increasing doses of X-ray irradiation (e.g., 0, 1, 2, 4, 6 and 8 Gy). This time, we exposed A549 and H1993 cells to conventional X-ray (0, 4, 8, 12 Gy; 3 Gy per min) emitted by a linear particle accelerator used for human radiotherapy operated at 6 MV and room temperature to obtain a proper radiation dose for our study.

### Clonogenic survival assays

Clonogenic survival assays described by Franken et al. [8] were used to evaluate the proliferative ability of irradiated A549 and H1993 cells. Briefly, cells were treated with either DMEM (control) or MβCD (5 or 10 mM) for 1 h followed by X-ray irradiation to a discontinuous rising dose of 0, 4, 8 and 12 Gy, and then cells were counted. Every 200 cells were seeded in a 35-mm dish at 37 °C under 5% carbon dioxide conditions and incubated for 30 days to allow macroscopic colony formation. Colonies were fixed with 4% paraformaldehyde for 20 min and then stained with Wright-Giemsa stain for 5 to 8 min. The number of colonies formed in each group was counted, and colonies containing approximately 50 viable cells were considered representative of clonogenic cells. The clonogenic fraction was calculated using these formulas: colony-plating efficiency (PE) = (number of colonies/number of seeded cells) × 100%; survival fraction (SF) = (PE of MβCD treated cells/PE of control cells) × 100%.

### Flow cytometry assays

Cell cycle and apoptosis analysis were performed with flow cytometry assays. Cells at a density of 2 × 10^6^/ml were exposed to either control DMEM or 5 or 10 mM MβCD for 1 h followed by X-ray irradiation (8 Gy) or control irradiation (0 Gy) then cultured in fresh DMEM. Cells were harvested and fixed in ice-cold 70% ethanol (4 °C) after being cultured for 4, 8, or 24 h. For cell cycle assays, after staining with 25 μl propidium iodide (PI, 100 μg/ml), the samples were incubated with 10 μl RNase A for 30 min in the dark at 37 °C. Cell apoptosis assays were performed with 5 μl PI for 15 min in the dark at 37 °C after mixing with 5 μl Annexin V-FITC. Cell cycle and apoptosis were evaluated by flow cytometry (C6; BD Biosciences, Franklin lakes, New Jersey, USA), and the data were analyzed with BD Accuri C6 Software 1.0.264.21.

### Immunofluorescence microscopy

Cells were plated on Lab-Tek chamber slides. After treatment with MβCD or control for 1 h followed by irradiation at 0 or 8 Gy, cells were fixed with 4% paraformaldehyde at 37 °C for 15 min, permeabilized with 0.5% Triton-X 100 after washing with PBS three times and then blocked with goat serum for 15 min. For lipid raft staining, cells were incubated with 0.05 mg/ml fluorescein isothiocyanate-conjugated-anti-cholera toxin subunit B for 1 h. For c-Met and c-Src staining, cells were incubated with anti-c-Met (Cy3-labeled) or anti-c-Src (Cy3-labeled) for 1 h then washed and blocked. 4′,6-Diamidine-2′-phenylindole dihydrochloride (DAPI) was used to stain the nuclei. Imaging was performed via fluorescence microscopy.

### Western immunoblotting analysis

Western immunoblotting analysis was performed as previously described [9]. Briefly, A549 cells were treated with indicated reagents (DMEM or 10 mM MβCD for 1 h followed by irradiation at 0 or 8 Gy) then washed with ice-cold PBS three times and lysed in RIPA lysis buffer containing 50 mM Tris-HCl (pH 7.4), 150 mM NaCl, 1% NP-40, and 0.1% SDS. Then, samples were centrifuged at 12000 rpm at 4 °C for 10 min in a low-temperature refrigerated centrifuge, and the supernatants were retained as protein lysates. For immunoblotting, 40 μg of protein lysates were subjected to electrophoresis on 4 to 10% SDS gels transferred to PVDF membranes, and blocked with 5% (*w*/*v*) skim milk in Tris-buffered saline-Tween 20 (0.05%, *v*/v; TTBS) for 1 h at 37 °C. Membranes were incubated overnight at 4 °C with primary antibodies against c-Met, p-c-Met, c-Src, p-c-Src and β-actin. After the overnight incubation, membranes were incubated with the appropriate horseradish peroxidase-conjugated specific goat anti-rabbit secondary antibody for 45 min and then washed with TTBS six times. The blots were developed by enhanced chemiluminescence followed by exposure to film, and the optical density values of target blots were analyzed with Gel-Pro-Analyzer software.

### Biochemical lipid raft isolation

**Fig. 1:**
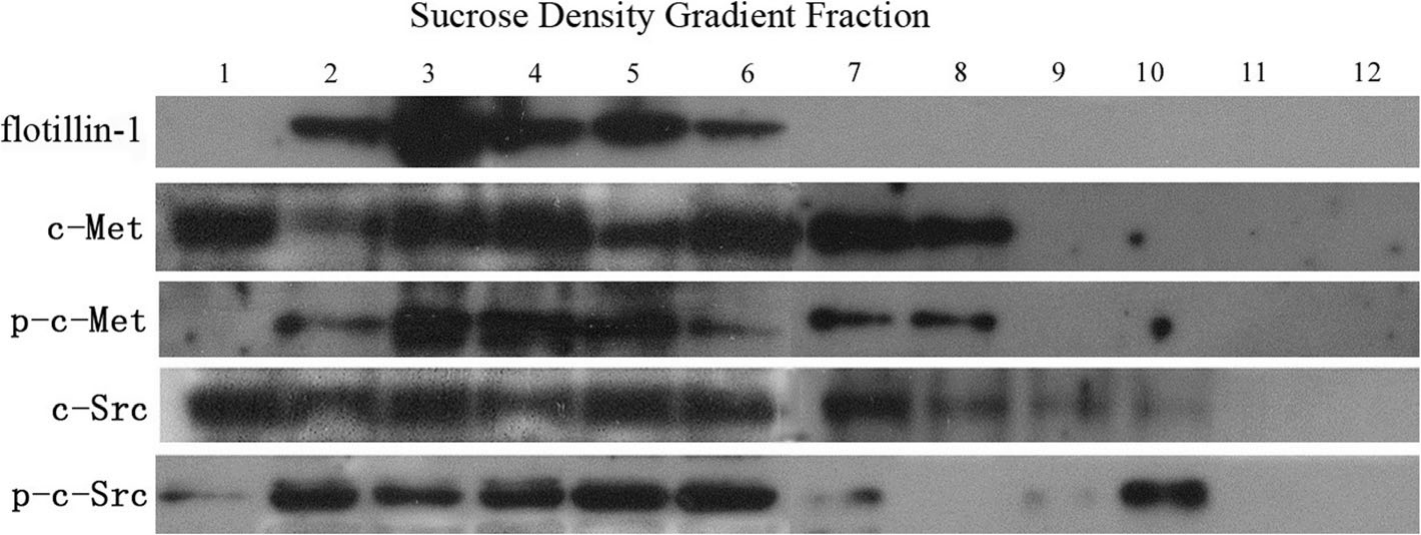
Lipid rafts were separated by a sucrose density gradient centrifugation procedure, and immunoblotting was performed for c-Met, p-c-Met, c-Src, p-c-Src and flotillin-1. Blots are representative of at least three independent experiments. Fractions 2–6 were determined to be lipid raft fractions due to the presence of the lipid raft-specific protein flotillin-1. c-Met was mainly distributed in fractions 1 and 3–8; p-c-Met was mainly distributed in fractions 2–8; c-Src was mainly distributed in fractions 1–8; and p-c-Src was mainly distributed in fractions 2–6 and 10

Biochemical lipid raft isolation was performed following established protocols [10, 11]. Briefly, all steps were performed at 4 °C. Cells were plated at a density of 1 × 10^7^ cells in six 100-mm plates. Treated and untreated cells were washed twice with cold PBS, scraped into 2 ml of TNE solution [0.5% Triton-X-100, 1 mM PMSF, 150 mM NaCl, and 1 mM EDTA] and incubated for 40 min. The samples were scraped and homogenized completely by passing through a 5-ml needle 40 times. Homogenates were mixed with 2 ml of 90% (*w*/*v*) sucrose and placed at the bottom of a 15-ml ultracentrifuge tube. A 5–35% (w/v) discontinuous sucrose gradient was formed above the homogenate-sucrose mixture with a 4-ml layer of 35% sucrose followed by a 4-ml layer of 5% sucrose by adding sucrose solution along the tube wall gently and slowly while avoiding any shake during the whole process. Next, samples were centrifuged at 39000 rpm at 4 °C for 20 h in a Beckman SW40 rotor. Twelve 1-ml gradient fractions were collected from the top of the gradient. Each fraction with no MβCD treatment and no irradiation was separated via SDS-PAGE and established the expression of flotillin-1, c-Met, p-c-Met, c-Src, p-c-Src by Western blot analysis. Fractions 2–6 were determined to be lipid raft fractions due to the presence of the lipid raft-specific protein flotillin-1 (Fig. 1). Then, we examined the total expression of c-Met, p-c-Met, c-Src, and p-c-Src in fractions 2–6 treated with either control DMEM or 10 mM MβCD for 1 h followed by irradiation to dose at 0 or 8 Gy.

### Statistical analysis

Student’s t-tests were performed utilizing the statistical software in GraphPad Prism version 5.0. Values of *P* < 0.05 were considered statistically significant. All the data expressed in our study are the mean ± SD from at least three independent experiments.

## Results

### MβCD suppressed proliferation of A549 and H1993 cells with or without X-ray irradiation

**Fig. 2:**
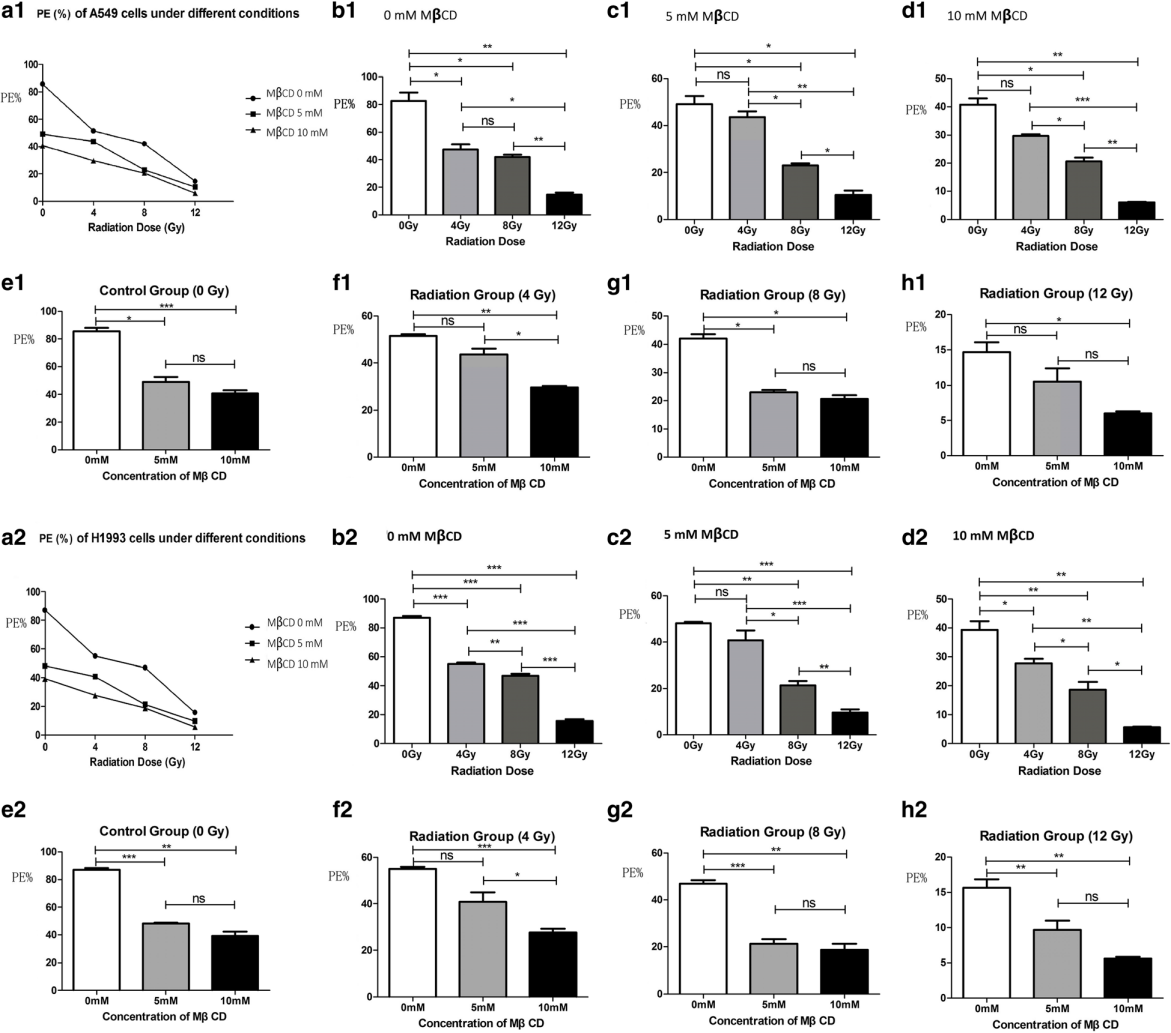
MβCD suppressed proliferation of A549 and H1993 cells whether followed by X-ray irradiation or not. Cells were pretreated with either control (DMEM) or MβCD (5 or 10 mM) for 1 h followed by X-ray irradiation to a discontinuous rising dose of 0, 4, 8 and 12 Gy. Then, cells were incubated for 30 days to allow macroscopic colony formation. The results showed that exposing A549 and H1993 cells to a rising dose of radiation either pretreated with DMEM or 5 or 10 mM MβCD inhibited cell proliferation in a radiation dose-dependent manner (**a1** represents PE(%) of A549 cells under different conditions. **b1-d1** represents PE(%) of A549 cells pretreated with the same concentration of MβCD (0, 5 or 10 mM, respectively) followed by different doses of X-ray(0, 4, 8 and 12 Gy). **e1-h1** represents PE(%) of A549 cells pretreated with different concentration of MβCD (0, 5 or 10 mM) followed by the same doses of X-ray(0, 4, 8 and 12 Gy, respectively). **a2** represents PE(%) of H1993 cells under different conditions. **b2-d2** represents PE(%) of H1993 cells pretreated with the same concentration of MβCD (0, 5 or 10 mM, respectively) followed by different doses of X-ray(0, 4, 8 and 12 Gy). **e2-h2** represents PE(%) of H1993 cells pretreated with different concentration of MβCD (0, 5 or 10 mM) followed by the same doses of X-ray(0, 4, 8 and 12 Gy, respectively). “no statistical significance” is shown as “ns”, “*P* < 0.05” is shown as “*”, “*P* < 0.01” is shown as “**”, and “*P* < 0.001” is shown as “***”)

MβCD was used to disrupt lipid rafts in cell membranes via depletion of cholesterol from the plasma membrane [12]. To assess a potential role for MβCD in suppressing proliferation, we exposed A549 and H1993 cells pretreated with either DMEM or MβCD to a rising dose of X-ray irradiation (0, 4, 8 and 12 Gy, respectively). The results of clonogenic survival assays were shown in Additional file 1: Table S1 and Additional file 2: Table S2 and Fig. 2. Within each cell line and each pretreatment group, there was a radiation dose-dependent decrease in colony-plating efficiency (PE) showing that higher radiation doses were significantly different from lower radiation doses except for A549 cells pretreated with DMEM followed by radiation of 4 Gy vs. 8 Gy, A549 cells pretreated with 5 mM MβCD followed by radiation of 0 Gy vs. 4 Gy, A549 cells pretreated with 10 mM MβCD followed by radiation of 0 Gy vs. 4 Gy, and H1993 cells pretreated with 5 mM MβCD followed by radiation of 0 Gy vs. 4 Gy. As shown in Additional file 1: Table S1 and Additional file 2: Table S2 and Fig. 2, the PEs of A549 and H1993 cells decreased in a radiation dose-dependent way. The PEs of A549 and H1993 cells in each group pretreated with the same concentration of MβCD irradiated with 8 Gy or 12 Gy X-ray compared with control group were significantly different. But this trend was not shown in each group irradiated with 4 Gy compared with control group. The PEs of A549 and H1993 cells pretreated with either DMEM or MβCD and irradiated with 12 Gy were too low to continue the remaining experiments; therefore, we chose 8 Gy as the proper radiation dose in our further experiments. Our results showed that the PEs of A549 cells in each group pretreated with 10 mM MβCD compared with DMEM followed by the same radiation dose (0, 4, 8 and 12 Gy, respectively) were significantly different, but the PEs were not significantly different in each radiation group pretreated with 5 mM MβCD vs. DMEM or 5 mM MβCD vs. 10 mM MβCD (Additional file 1: Table S1 and Fig. 2). This trend was also shown in H1993 cells (Additional file 2: Table S2 and Fig. 2). Therefore, we chose 10 mM as the proper concentration of MβCD for our further experiments. These results showed that MβCD suppressed the proliferation of A549 and H1993 cells whether followed by X-ray irradiation or not.

### The combined treatment of MβCD and radiation resulted in additive increases in apoptosis of A549 and H1993 cells

**Fig. 3:**
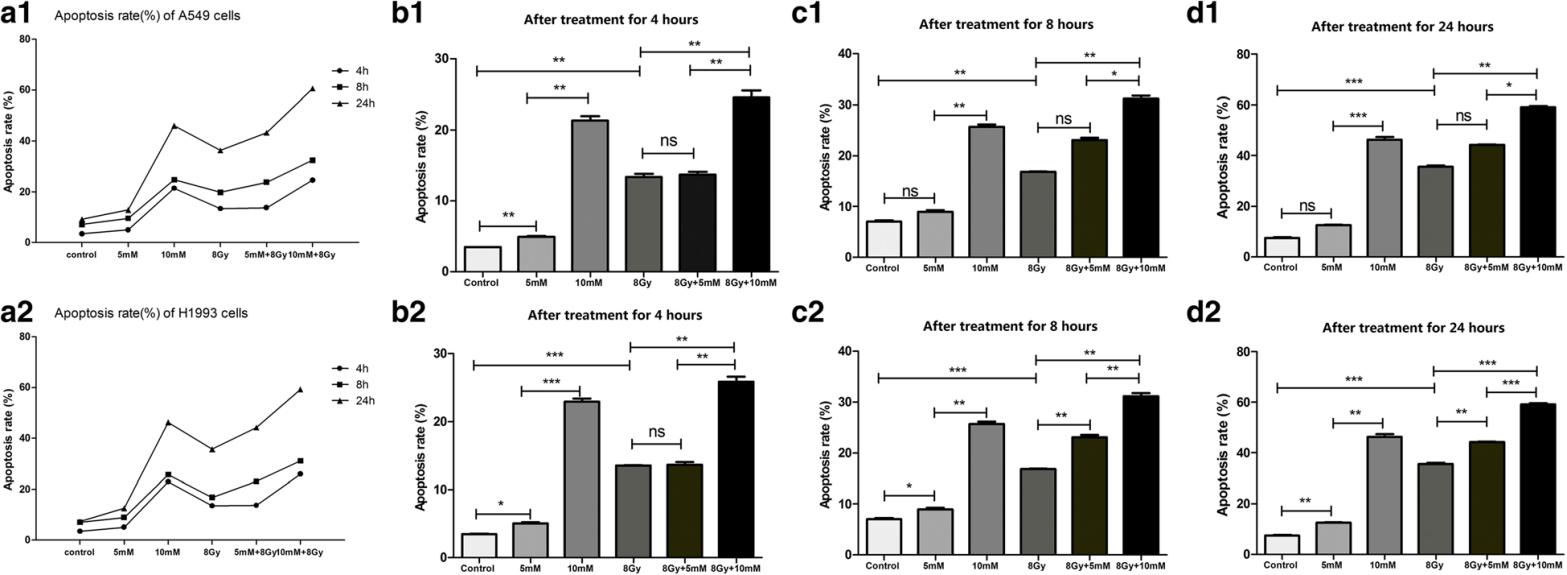
The apoptosis rate of A549 and H1993 cells in each group pretreated with 10 mM MβCD compared with DMEM followed by the same radiation dose were significantly different but not significantly different in each group pretreated with 5 mM MβCD vs. DMEM or 5 mM MβCD vs. 10 mM MβCD (**a1** represents the apoptosis rate of A549 cells under different conditions. **b1-d1** represents the apoptosis rate of A549 cells after treatment for 4, 8, 24 hours respectively. **a2** represents the apoptosis rate of H1993 cells under different conditions. **b2-d2** represents the apoptosis rate of H1993 cells after treatment for 4, 8, 24 hours respectively. “no statistical significance” is shown as “ns”, “*P* < 0.05” is shown as “*”, “*P* < 0.01” is shown as “**”, and “*P* < 0.001” is shown as “***”)

In this study, we aimed to ascertain whether the combination of MβCD and radiation had an additive or supra-additive effect on the apoptosis of A549 and H1993 cells. Notably, pretreatment with 5 mM MβCD alone produced little apoptosis in both A549 and H1993 cells (Fig. 3). Increasing the concentration of MβCD to 10 mM greatly increased the apoptosis rate in both cell lines (Fig. 3). The combined treatment of 5 mM MβCD and X-ray irradiation (8 Gy) did not significantly differ from that of X-ray irradiation alone at 4, 8, or 24 h in A549 cells or at 4 h in H1993 cells with respect to an additive effect on apoptosis (at 4, 8 and 24 h in A549 cells: *P* = 0.1124, *P* = 0.0650, *P* = 0.1110; at 4 h in H1993 cells: *P* = 0.7438; respectively; Fig. 3); however, the combined treatment of 5 mM MβCD and X-ray irradiation (8 Gy) significantly increased apoptosis at 8 and 24 h in H1993 cells (at 8 h and 24 h: *P* = 0.0071, *P* = 0.0010, respectively; Fig. 3). The combination of 10 mM MβCD and radiation (8 Gy) markedly increased the apoptosis rate when compared with that of radiation alone at 4, 8 and 24 h, and the differences were statistically significant (at 4, 8 and 24 h in A549 cells: *P* = 0.0026, *P* = 0.0013, and *P* = 0.0016; at 4, 8 and 24 h in H1993 cells: *P* = 0.0038, *P* = 0.0020, and *P* = 0.0002, respectively; Fig. 3). These results showed that the combination of MβCD and radiation resulted in additive increases in the apoptosis of A549 and H1993 cells.

### X-ray irradiation induced the redistribution of c-met and c-Src in lipid rafts

**Fig. 4:**
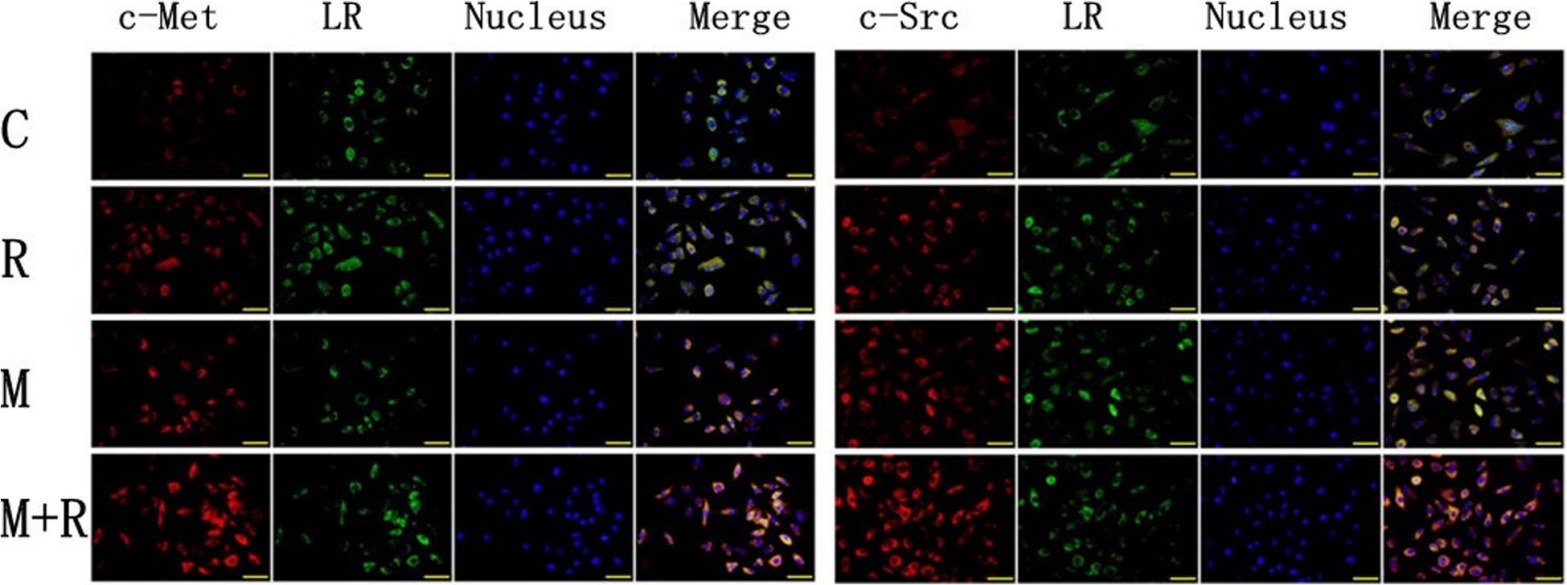
X-ray irradiation induced the aggregation of lipid rafts and clustering of c-Met and c-Src to lipid rafts in A549 cells. MβCD blocked both the aggregation of lipid rafts and clustering of c-Met and c-Src to lipid rafts(C: control group, R: radiation only group, M: MβCD only group, M + R: MβCD and radiation combined group, LR: lipid raft marker)

To investigate the impact of X-ray irradiation on the redistribution of c-Met and c-Src in lipid rafts, A549 cells were treated with 10 mM MβCD or control (DMEM) for 1 h followed by X-ray irradiation to a dose of 0 or 8 Gy. Sixteen hours later, the distribution of c-Met and c-Src in lipid rafts was determined (Fig. 4). The results showed that X-ray irradiation alone induced the aggregation of lipid rafts and clustering of c-Met and c-Src to lipid rafts. Through destroying the integrity of lipid rafts, MβCD pretreatment blocked both the aggregation of lipid rafts and clustering of c-Met and c-Src to lipid rafts.

### The activation of c-met and c-Src and the accumulation of c-met and c-Src to lipid rafts were restrained by MβCD

In agreement with the sucrose density gradient centrifugation procedure [10, 11], the original location of c-Met, p-c-Met, c-Src and p-c-Src in A549 cells with no MβCD treatment and no X-ray irradiation was revealed (Fig. 1): c-Met was mainly distributed in fractions 1 and 3–8; p-c-Met was mainly distributed in fractions 2–8; c-Src was mainly distributed in fractions 1–8; and p-c-Src was mainly distributed in fractions 2–6 and 10. As we mentioned before, fractions 2–6 were determined to be the lipid raft fractions.

**Fig. 5:**
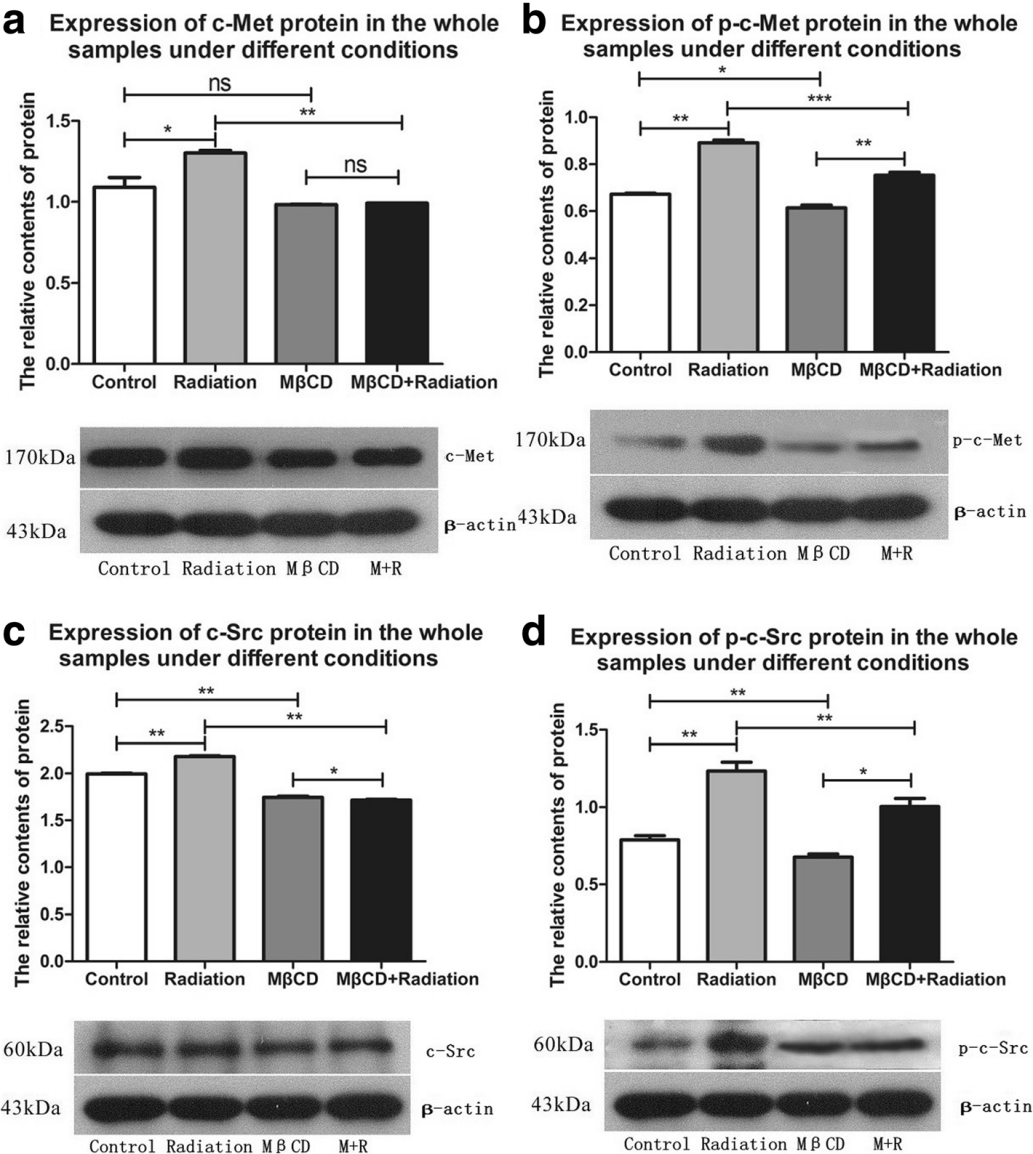
Expression of c-Met, p-c-Met, c-Src and p-c-Src in the whole-cell samples was significantly increased by X-ray irradiation in A549 cells. However, this up-regulation was blocked by pretreatment with MβCD (**a-d** presents the expression of c-Met, p-c-Met, c-Src and p-c-Src in the whole-cell samples under different conditions respectively. “no statistical significance” is shown as “ns”, “*P* < 0.05” is shown as “*”, “*P* < 0.01” is shown as “**”, and “*P* < 0.001” is shown as “***”)

**Fig. 6:**
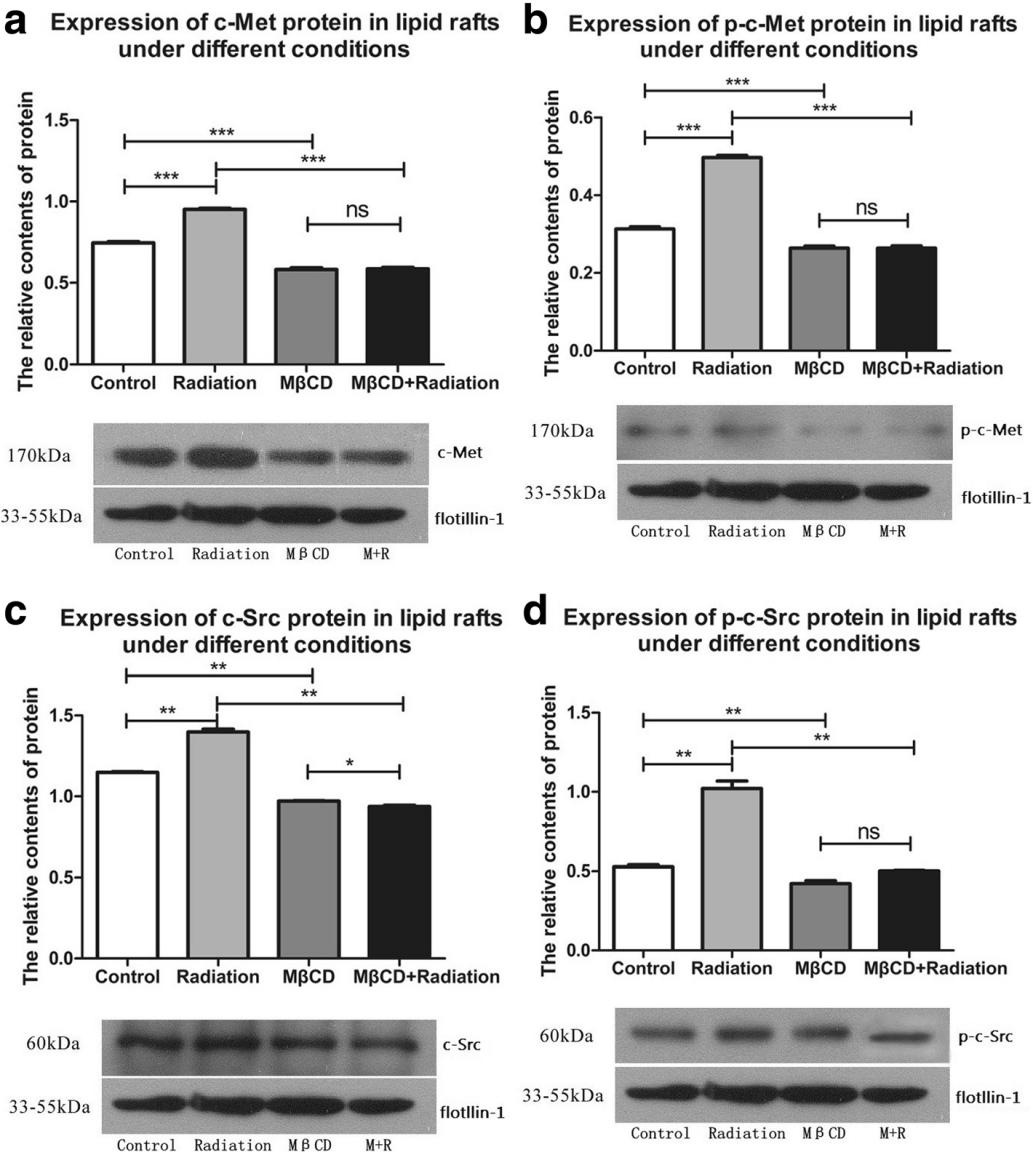
Expression of c-Met, p-c-Met, c-Src and p-c-Src in lipid rafts was significantly increased by X-ray irradiation in A549 cells. However, this up-regulation was blocked by pretreatment with MβCD (**a-d** presents the expression of c-Met, p-c-Met, c-Src and p-c-Src in lipid rafts under different conditions respectively. “no statistical significance” is shown as “ns”, “*P* < 0.05” is shown as “*”, “*P* < 0.01” is shown as “**”, and “*P* < 0.001” is shown as “***”)

The expression levels of c-Met, p-c-Met (activated c-Met), c-Src and p-c-Src (activated c-Src) in the whole-cell samples were significantly increased after X-ray irradiation (c-Met, p-c-Met, c-Src, and p-c-Src: *P* = 0.0406, *P* = 0.0012, *P* = 0.0085, and *P* = 0.0045, respectively; Additional file 3: Table S3 and Fig. 5) when compared with those of the control group. However, this up-regulation of c-Met, p-c-Met, c-Src and p-c-Src in the whole-cell samples was blocked by pretreatment with MβCD (c-Met, p-c-Met, c-Src, and p-c-Src: *P* = 0.0033, *P* = 0.0005, *P* = 0.0012, and *P* = 0.0024, respectively; Additional file 3: Table S3 and Fig. 5). The sucrose density gradient centrifugation results showed that the accumulation of c-Met, p-c-Met, c-Src and p-c-Src to lipid rafts was significantly induced by X-ray irradiation (c-Met, p-c-Met, c-Src, and p-c-Src: *P* < 0.0001, *P* < 0.0001, *P* = 0.0030, and *P* = 0.0051, respectively; Additional file 4: Table S4 and Fig. 6). Moreover, this accumulation of c-Met, p-c-Met, c-Src and p-c-Src to lipid rafts was blocked by pretreatment with MβCD (c-Met, p-c-Met, c-Src, and p-c-Src: *P* < 0.0001, *P* < 0.0001, *P* = 0.0028, and *P* = 0.0082, respectively; Additional file 4: Table S4 and Fig. 6).

**Fig. 7:**
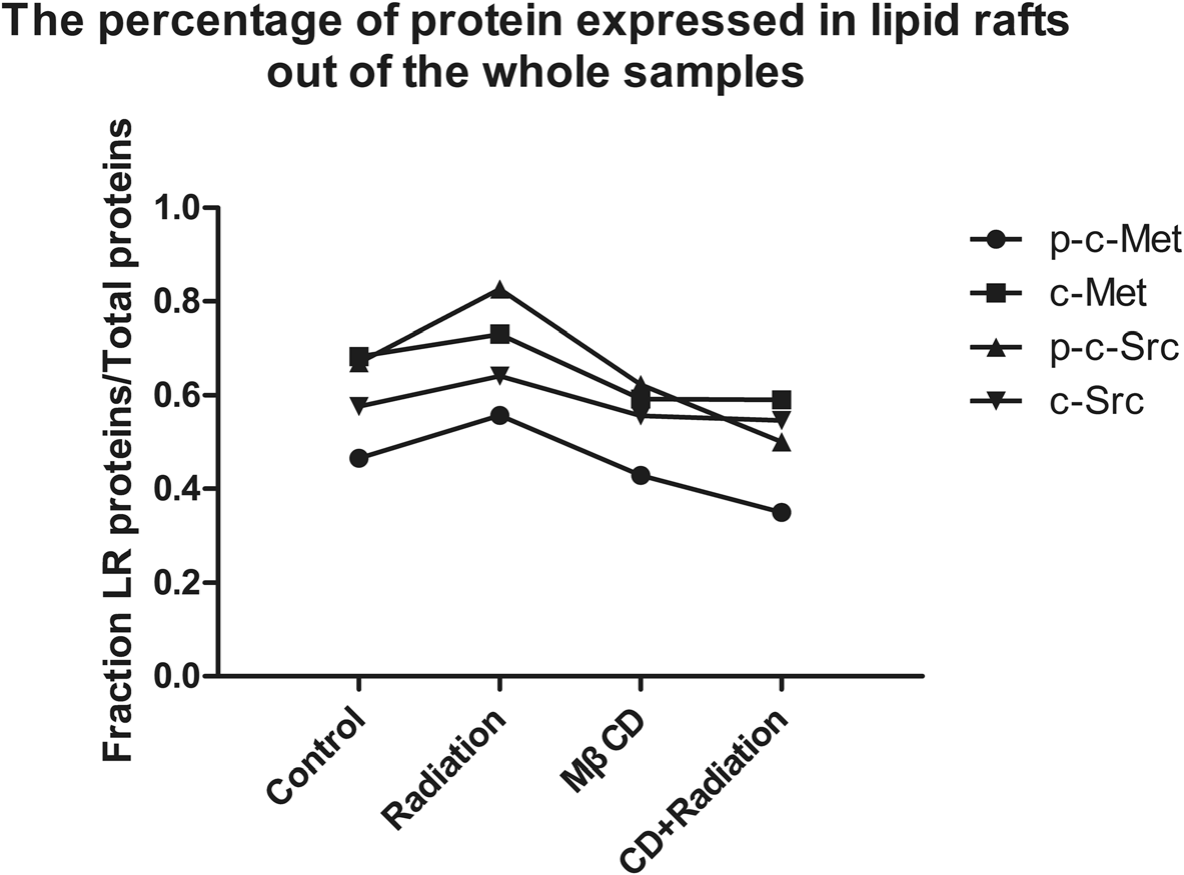
The percentages of c-Met, p-c-Met, c-Src and p-c-Src expressed in lipid rafts out of the whole samples in A549 cells

Interestingly, compared with the MβCD alone group, the combined treatment group showed significantly increased expression of p-c-Met and p-c-Src in the whole-cell samples. However, there was no significant change in the accumulation of p-c-Met or p-c-Src to lipid rafts. More importantly, the percentages of p-c-Met and p-c-Src expressed in lipid rafts out of those expressed in the whole-cell samples were obviously decreased in the combined group when compared with the MβCD alone group (Additional file 5: Table S5 and Fig. 7).

## Discussion

Lung cancer is the leading cause of cancer death worldwide, and NSCLC accounts for approximately 85% of the total number of lung cancer diagnoses. Although significant progress in diagnosis and treatment has been made over the past several years, it is still too late for most NSCLC patients to have radical surgery at their first diagnosis. Radiotherapy is one of the basic treatments for unresectable NSCLC, but the resistance to radiation greatly limits the curative effect of radiotherapy. Currently, cellular survival pathways that regulate DNA damage repair after radiotherapy have been heavily researched to reveal the mechanism of NSCLC radiation resistance [13]. Many clinical studies have shown that the radiotherapy resistance of various solid tumors is associated with the overexpression of c-Met [14–16]. c-Met is a 170-kDa transmembrane protein that can be activated by binding hepatocyte growth factor (HGF) to its extracellular region [17]. De Bacco et al. demonstrated that irradiation directly induced the overexpression and activity of the Met oncogene and activated c-Met signaling through the ATM-NF-κB signaling pathway. In turn, the activated c-Met signaling triggered the activation of downstream signaling, mainly through the PI3K/Akt, MAPK, and STAT pathways [18]. The activation of c-Met and its downstream signaling pathways has been shown to induce invasion and migration of cancer cells [19]. Fan et al. showed that the activation of c-Met protected tumor cells from DNA damage caused by radiation and led to radiation resistance [20]. Overexpression of c-Met has been noted in various tumors, and c-Met activation appears to be associated with increased tumor differentiation, shorter survival times and an overall worse prognosis in patients with NSCLC [21, 22]. c-Src, a non-receptor tyrosine kinase, is localized to intracellular membranes. c-Src is overexpressed or highly activated in a number of human malignancies, including carcinomas of the breast, lung, colon, esophagus, skin, parotid, cervix, and gastric tissues, as well as in the development of cancer and progression to distant metastases [23]. Recent studies have shown that c-Src enhances DNA damage repair and induces NSCLC radiation resistance through ERK, AKT, and NF-κB pathways [13]. c-Met activates the PI3K/Akt, ERK, and NF-κB pathways via c-Src in cervical cancer cells [24–26]. c-Src might be the bridge by which the c-Met signaling pathway induces radiation resistance.

The plasma membrane is the structural basis for signal transduction. Lipid rafts are small (10–200 nm), heterogeneous, highly dynamic, sterol- and sphingolipid-enriched domains that compartmentalize cellular processes. Smaller lipid rafts can stabilize their structure and form a larger platform through protein-protein and protein-lipid interactions [27]. As a “highly dynamic platform”, the lipid raft environment plays an important role in cell proliferation, apoptosis, and functional activities through regulating various cell signal transduction mechanisms. Hanahan et al. summarized that the occurrence and development of tumors is closely connected with uncontrolled cell proliferation, resisting apoptosis, evading growth suppressors, enabling replicative immortality, inducing angiogenesis, activating invasion and metastasis, reprogramming of energy metabolism and evading immune destruction [28]. A growing body of evidence has shown that lipid raft microdomains provide signaling platforms that regulate a variety of cellular signaling pathways through which tumors can be initiated and developed [29–31]. Recent studies have shown that the activated residual of c-Met located in lipid rafts, which serve as a huge signaling platform for the activation of c-Met and its downstream pathways [3]. Localization of c-Src to lipid rafts has been demonstrated in a variety of cancer cell lines [32]. We hypothesized that disturbing the integrity of lipid rafts would block the activation of the c-Met signaling pathway and reverse the radiation resistance of NSCLC cells in some way.

In this study, the clonogenic survival assays showed that X-ray irradiation inhibited the proliferation of A549 and H1993 cells in a radiation dose-dependent manner regardless of MβCD pretreatment. Our results further confirmed that inhibiting the integrity of lipid rafts suppressed the proliferation of A549 and H1993 cells whether followed by X-ray irradiation or not. Furthermore, we found the proper concentration of MβCD (10 mM) and the proper radiation dose (8 Gy) for our remaining experiments.

Next, we found that pretreating A549 and H1993 cells with 10 mM MβCD alone obviously increased the apoptosis rate in both control (0 Gy) and irradiated cells (8 Gy) but not for 5 mM MβCD alone. Our results also showed that the combined treatment of MβCD and radiation significantly increased the apoptosis rates of A549 and H1993 cells when compared with those of radiation alone at 4, 8 and 24 h, but this effect was not significant for the combination of 5 mM MβCD and radiation (8 Gy) compared with radiation alone. These results suggest that disturbing the integrity of lipid rafts by MβCD sensitized A549 and H1993 cells to radiotherapy in both time-dependent and concentration-dependent manners. Our findings also indicate that lipid rafts play an important role in increasing the radiation sensitivity of NSCLC cells, and the combination of MβCD and radiation may provide a new effective therapeutic strategy for the treatment of radiation-resistant NSCLC.

To investigate the impact of X-ray irradiation on the redistribution of c-Met and c-Src in lipid rafts, A549 cells were treated with 10 mM MβCD or DMEM for 1 h followed by X-ray irradiation to a dose of 0 or 8 Gy, and the distribution of c-Met and c-Src in lipid rafts was determined 16 h later. The results showed that X-ray irradiation induced the aggregation of lipid rafts and the clustering of c-Met and c-Src to lipid rafts. The results also demonstrated that destroying the integrity of lipid rafts restrained both the aggregation of lipid rafts and the clustering of c-Met and c-Src to lipid rafts. These results indicate that X-ray irradiation-induced redistribution of c-Met and c-Src in lipid rafts might result in radiation resistance in NSCLC cells.

Western blotting results showed that X-ray irradiation significantly increased the expression of c-Met, p-c-Met, c-Src and p-c-Src in the whole-cell samples, but this up-regulation was blocked by pretreatment with MβCD. The sucrose density gradient centrifugation analysis showed that X-ray irradiation significantly induced the accumulation of c-Met, p-c-Met, c-Src and p-c-Src to lipid rafts. Furthermore, the accumulation of these four proteins to lipid rafts was blocked by pretreatment with MβCD. Interestingly, we also found that the expression levels of p-c-Met and p-c-Src in the whole-cell samples were significantly increased in the combined group compared with those in the MβCD alone group. However, there was no significant change in the accumulation of these two proteins to lipid rafts. The percentages of p-c-Met and p-c-Src expressed in lipid rafts out of the whole-cell samples was obviously decreased in the combined treatment group when compared with those in the MβCD alone group. Collectively, these results show that X-ray irradiation might activate c-Met and c-Src through both lipid raft-dependent and lipid raft-independent mechanisms.

By analyzing the percentages of c-Met, p-c-Met, c-Src, and p-c-Src proteins expressed in lipid rafts out of the whole-cell samples, we found that in A549 cells, the expression of p-c-Met and p-c-Src in lipid rafts induced by X-ray irradiation was significantly higher than that of c-Met and c-Src. Furthermore, the inhibition of p-c-Met and p-c-Src expressed in lipid rafts was more obvious than that of c-Met and c-Src by the destruction of lipid rafts.

In summary, this study confirmed that MβCD suppressed the proliferation of human NSCLC cell lines A549 and H1993 with or without X-ray irradiation, and the combination of MβCD and radiation resulted in additive increases in the apoptosis of A549 and H1993 cells. X-ray irradiation induced the aggregation of lipid rafts and the clustering of c-Met and c-Src to lipid rafts through both lipid raft-dependent and lipid raft-independent mechanisms. Our results also demonstrated that destroying the integrity of lipid rafts significantly inhibited the aggregation of c-Met and c-Src to lipid rafts. More importantly, the expression of p-c-Met and p-c-Src in lipid rafts induced by X-ray irradiation was notably higher than that of c-Met and c-Src. Furthermore, the inhibition of p-c-Met and p-c-Src expressed in lipid rafts was more obvious than that of c-Met and c-Src by destruction of lipid rafts.

## Conclusions

Taken together, we draw a conclusion that lipid rafts serve as the signaling platforms for the lipid raft-dependent activation of c-Met and c-Src induced by X-ray irradiation. The lipid raft-dependent activation of c-Met and its downstream pathways play an important role in radiation resistance of NSCLC cells mediated by c-Met. Destroying the integrity of lipid rafts can reverse these signaling pathways and improve the radiosensitivity of NSCLC cells, which can provide a new strategy for developing radiation sensitizing agents and for improving the therapeutic effect of radiotherapy. Further studies are still required to explore the molecular mechanisms of the activation of c-Met and c-Src in lipid rafts induced by radiation.

## Additional files

Additional file 1: **Table S1.** Colony-plating efficiency (PE) of A549 cells treated with either control or MβCD followed by irradiation. (DOC 28 kb)

Additional file 2: **Table S2.** Colony-plating efficiency (PE) of H1993 cells treated with either control or MβCD followed by irradiation. (DOC 29 kb)

Additional file 3: **Table S3.** Expression of proteins in the whole-cell samples under different conditions in A549 cells. (DOC 28 kb)

Additional file 4: **Table S4.** Expression of proteins in lipid rafts under different conditions in A549 cells. (DOC 28 kb)

Additional file 5: **Table S5.** The percentage of protein expressed in lipid rafts out of the whole-cell samples in A549 cells. (DOC 28 kb)

## Abbreviations

c-Met: Mesenchymal-epithelial transition factor
DAPI: 4′,6-Diamidine-2′-phenylindole dihydrochloride
HGF: Hepatocyte growth factor
MβCD: Methyl-β-cyclodextrin
NSCLC: Non-small cell lung cancer
PE: Colony-plating efficiency
PI: Propidium iodide
PMSF: Phenylmethanesulfonyl fluoride
RIPA: Radioimmunoprecipitation assay
SF: Survival fraction
