# Supplementary material for: Aggregation of lipid rafts activates c-met and c-Src in non-small cell lung cancer cells
Source: BMC Cancer. 2018 May 30;18:611. doi: 10.1186/s12885-018-4501-8 (PMC5977465; doi:10.1186/s12885-018-4501-8)
Supplement: Supplementary file 1 — Table S1. Colony-plating efficiency (PE) of A549 cells treated with either control or MβCD followed by irradiation. (DOC 28 kb) [file 12885_2018_4501_MOESM1_ESM.doc]

Table 1. Colony-plating efficiency (PE) of A549 cells treated with either control or MβCD followed by irradiation

| Radiation MβCD | 0 mM | 5 mM | 10 mM |
| --- | --- | --- | --- |
| 0 Gy | 85.67±4.25% | 49.17±5.97% | 40.83±3.79% |
| 4 Gy | 51.50±1.32% | 43.67±4.19% | 29.67±1.04% |
| 8 Gy | 42.00±2.65% | 23.00±1.50% | 20.67±2.36% |
| 12 Gy | 14.67±2.47% | 10.50±3.28% | 6.00±0.50% |
