# Supplementary material for: Aggregation of lipid rafts activates c-met and c-Src in non-small cell lung cancer cells
Source: BMC Cancer. 2018 May 30;18:611. doi: 10.1186/s12885-018-4501-8 (PMC5977465; doi:10.1186/s12885-018-4501-8)
Supplement: Supplementary file 2 — Table S2. Colony-plating efficiency (PE) of H1993 cells treated with either control or MβCD followed by irradiation. (DOC 29 kb) [file 12885_2018_4501_MOESM2_ESM.doc]

Table 2. Colony-plating efficiency (PE) of H1993 cells treated with either control or MβCD followed by irradiation

| Radiation MβCD | 0 mM | 5 mM | 10 mM |
| --- | --- | --- | --- |
| 0 Gy | 87.00±2.29% | 48.17±1.04% | 39.33±5.13% |
| 4 Gy | 55.00±1.50% | 40.77±7.28% | 27.67±2.75% |
| 8 Gy | 46.97±2.45% | 21.33±3.25% | 18.67±4.64% |
| 12 Gy | 15.67±2.02% | 9.67±2.26% | 5.60±0.46% |
