# Supplementary material for: Aggregation of lipid rafts activates c-met and c-Src in non-small cell lung cancer cells
Source: BMC Cancer. 2018 May 30;18:611. doi: 10.1186/s12885-018-4501-8 (PMC5977465; doi:10.1186/s12885-018-4501-8)
Supplement: Supplementary file 3 — Table S3. Expression of proteins in the whole-cell samples under different conditions in A549 cells. (DOC 28 kb) [file 12885_2018_4501_MOESM3_ESM.doc]

Table 3. Expression of proteins in the whole-cell samples under different conditions in A549 cells

| Groups | p-c-Met | c-Met | p-c-Src | c-Src |
| --- | --- | --- | --- | --- |
| C | 0.6718±0.0078 | 1.0912±0.1036 | 0.7879±0.0490 | 1.9951±0.0152 |
| R | 0.8910±0.0209 | 1.3021±0.0289 | 1.2340±0.0995 | 2.1812±0.0147 |
| M | 0.6137±0.0210 | 0.9825±0.0062 | 0.6766±0.0351 | 1.7451±0.0231 |
| M+R | 0.7531±0.0213 | 0.9915±0.0023 | 1.0041±0.0919 | 1.7150±0.0147 |
