# Supplementary material for: Aggregation of lipid rafts activates c-met and c-Src in non-small cell lung cancer cells
Source: BMC Cancer. 2018 May 30;18:611. doi: 10.1186/s12885-018-4501-8 (PMC5977465; doi:10.1186/s12885-018-4501-8)
Supplement: Supplementary file 4 — Table S4. Expression of proteins in lipid rafts under different conditions in A549 cells. (DOC 28 kb) [file 12885_2018_4501_MOESM4_ESM.doc]

Table 4. Expression of proteins in lipid rafts under different conditions in A549 cells

| Groups | p-c-Met | c-Met | p-c-Src | c-Src |
| --- | --- | --- | --- | --- |
| C | 0.3127±0.0100 | 0.7454±0.0143 | 0.5274±0.0203 | 1.1491±0.0065 |
| R | 0.4965±0.0099 | 0.9513±0.0113 | 1.0201±0.0816 | 1.3980±0.0294 |
| M | 0.2632±0.0101 | 0.5821±0.0176 | 0.4216±0.0334 | 0.9701±0.0062 |
| M+R | 0.2634±0.0098 | 0.5858±0.0156 | 0.5023±0.0047 | 0.9371±0.0135 |
