# Supplementary material for: Aggregation of lipid rafts activates c-met and c-Src in non-small cell lung cancer cells
Source: BMC Cancer. 2018 May 30;18:611. doi: 10.1186/s12885-018-4501-8 (PMC5977465; doi:10.1186/s12885-018-4501-8)
Supplement: Supplementary file 5 — Table S5. The percentage of protein expressed in lipid rafts out of the whole-cell samples in A549 cells. (DOC 28 kb) [file 12885_2018_4501_MOESM5_ESM.doc]

Table 5. The percentage of protein expressed in lipid rafts out of the whole-cell samples in A549 cells

| Groups | c-Met | p-c-Met | c-Src | p-c-Src |
| --- | --- | --- | --- | --- |
| C | 68.32% | 46.55% | 57.59% | 66.94% |
| R | 73.06% | 55.72% | 64.10% | 82.66% |
| M | 59.24% | 42.89% | 55.59% | 62.31% |
| M+R | 59.08% | 34.98% | 54.64% | 50.03% |
